# Inflammatory and cytotoxic effects of bifenthrin in primary microglia and organotypic hippocampal slice cultures

**DOI:** 10.1186/s12974-018-1198-1

**Published:** 2018-05-24

**Authors:** Brahim Gargouri, Nizar M. Yousif, Michèle Bouchard, Hamadi Fetoui, Bernd L. Fiebich

**Affiliations:** 1 Neuroimmunology and Neurochemistry Research Group, Department of Psychiatry and Psychotherapy, Medical Center – University of Freiburg, Faculty of Medicine, University of Freiburg, Freiburg, Germany; 2 Laboratory of Toxicology-Microbiology and Environmental Health (17ES06), Sciences Faculty of Sfax, University of Sfax, BP1171, 3000 Sfax, Tunisia; 3 Department of Environmental and Occupational Health, Toxicological Risk Assessment and Management, University of Montreal, Roger-Gaudry Building, U424, Main Station, Montreal, P.O. Box 6128, Montreal, Quebec H3C 3J7 Canada; 4 Faculty of Biology, University of Freiburg, Freiburg, Germany; 5 Laboratory of Translational Psychiatry, Department of Psychiatry and Psychotherapy, Medical Center Faculty of Medicine, University of Freiburg, Hauptstrasse 5, 79104 Freiburg, Germany

**Keywords:** Bifenthrin, Microglia, Oxidative stress, OHSCs, Nrf-2, NF-kappaB

## Abstract

**Background:**

Pyrethroids, such as bifenthrin (BF), are among the most widely used class of insecticides that pose serious risks to human and wildlife health. Pyrethroids are proposed to affect astrocytic functions and to cause neuron injury in the central nervous system (CNS). Microglia are key cells involved in innate immune responses in the CNS, and microglia activation has been linked to inflammation and neurotoxicity. However, little information is known about the effects of BF-induced toxicity in primary microglial cells as well as in organotypic hippocampal slice cultures (OHSCs).

**Methods:**

Oxidative stress and inflammatory responses induced by BF were evaluated in primary microglial cells and OHSCs incubated with different concentrations of BF (1–20 μM) for 4 and 24 h. mRNA and protein synthesis of cyclooxygenase-2 (COX-2), tumor necrosis factor-alpha (TNF-alpha), interleukin-6 (IL-6), nuclear erythroid-2 like factor-2 (Nrf-2), and microsomal prostaglandin synthase-1 (mPGES-1) was also studied by qPCR and Western blot. Cell viability was analyzed by MTT-tetrazolio (MTT) and lactate dehydrogenase (LDH) assays. Neurotoxicity in OHSCs was analyzed by propidium iodide (PI) staining and confocal microscopy.

**Results:**

Exposure of microglial cells to BF for 24 h resulted in a dose-dependent reduction in the number of viable cells. At sub-cytotoxic concentrations, BF increased reactive oxygen species (ROS), TNF-alpha synthesis, and prostaglandin E_2_ (PGE_2_) production, at both 4- and 24-h time points, respectively. Furthermore, BF incubation decreased superoxide dismutase (SOD), catalase (CAT), and glutathione peroxidase (GPx) activities and increased lipid peroxidation, protein oxidation, and H_2_O_2_ formation. In addition, BF significantly induced protein synthesis and mRNA expression of oxidative and inflammatory mediators after 4 and 24 h, including Nrf-2, COX-2, mPGES-1, and nuclear factor kappaB (NF-kappaB). A 24-h exposure of OHSCs to BF also increased neuronal death compared to untreated controls. Furthermore, depletion of microglia from OHSCs potently enhanced neuronal death induced by BF.

**Conclusions:**

Overall, BF exhibited cytotoxic effects in primary microglial cells, accompanied by the induction of various inflammatory and oxidative stress markers including the Nrf-2/COX-2/mPGES-1/NF-kappaB pathways. Moreover, the study provided evidence that BF induced neuronal death in OHSCs and suggests that microglia exert a protective function against BF toxicity.

**Electronic supplementary material:**

The online version of this article (10.1186/s12974-018-1198-1) contains supplementary material, which is available to authorized users.

## Background

Extensive application of pesticides is usually accompanied by serious pollution problems and health hazards. Today, their extensive occupational and domestic use raises many questions about their deleterious health effects [1]. Synthetic pyrethroids (SPs) are among the most commonly used classes of pesticides in agricultural and household formulations and account for one fourth of the total insecticide market worldwide [2–5]. Most importantly, SPs are often classified into two groups based on their chemical structures and neurotoxicological effect. Type I pyrethroids, which lack an alpha-cyano moiety, induce a syndrome consisting of aggressive sparring and fine tremor progressing to whole-body tremor and prostration in rats (T-syndrome). Type II pyrethroids, which contain an alpha-cyano moiety, produce a syndrome that includes pawing, burrowing, salivation, and coarse tremors leading to choreoathetosis in rats (CS-syndrome) [6, 7]. Although this classification is useful in characterizing these chemicals, a few pyrethroids elicit neurotoxic signs of both syndromes [6, 8, 9]. Furthermore, this classification is based on high-dose effects and may not relate to low-dose effects of SPs [8, 10, 11].

SPs, including bifenthrin (BF), have been found to induce oxidative stress in animal models resulting in both increased oxidant markers and decreased antioxidant activities [12, 13]. BF is a type I pyrethroid insecticide that can cross the blood-brain barrier [11, 14] and induces neurotoxicity by prolonging the opening of voltage-gated sodium channels (VGSC) [8, 15]. This prolonged opening of Na^+^ channels results in persistent depolarization leading to repetitive firing, and if the exposure is high enough, it can lead to seizures, paralysis, and death [16]. However, recent attention has focused on potential alternative targets that may be involved in pyrethroid toxicity [17]. Specifically, various lines of evidence have suggested possible roles for nuclear erythroid-2 like factor-2 (Nrf-2) and nuclear factor kappaB (NF-kappaB) pathways in the acute manifestation of neurotoxicity and oxidative stress elicited by pyrethroids and some food contaminants such as acrylamide (AA) [9, 18, 19]. In addition, exposure to type I pyrethroids has been shown to induce the release of intracellular Ca^2+^ and reactive oxygen species (ROS) which can lead to DNA damage and cell death. This cascade involves the activation of NF-kappaBp65, cyclooxygenase-2 (COX-2), microsomal prostaglandin synthase-1 (mPGES-1), and Nrf-2 in the brains of mice and rats [19, 20]. There is also rising evidence that long-term/low-dose SP exposure may have significant neurotoxic effects [21]. In fact, nigrostriatal dopaminergic neurodegeneration has been reported in adult rats following 12 weeks of cypermethrin (CM) exposure [14, 22]. Furthermore, exposure to the type II pyrethroid deltamethrin (DM) was shown to induce apoptosis both in vitro and in vivo in a mouse model [23].

Microglia are macrophage-like resident cells of the CNS [24]. Excessive inflammation involving microglia activation is seen in many neurodegenerative diseases such as Parkinson’s disease, Alzheimer’s disease (AD), and multiple sclerosis (MS) [25–29]. Upon activation, microglia release inflammatory cytokines and increase the production of inducible nitric oxide synthase (iNOS) and ROS, two oxidative stress markers that have been associated with demyelination and axonal damage in cerebellar cultures [30]. On the other hand, emerging evidence also suggests that activated microglia might act as effectors of processes that promote tissue recovery under pathological conditions [31]. More recently, several pesticides (e.g., permethrin (PM), deltamethrin (DM), and allethrin (AL)) were shown to directly induce the production of inflammatory mediators by microglia [32, 33]. Indeed, inflammatory mediators generated by innate immune CNS cells such as microglia are considered the major culprits in neurodegenerative diseases [34, 35].

Like other cell types, microglia express the Nrf-2 and heme oxygenase-1 (HO-1) systems, which are essential for counteracting oxidative stress and inflammation [36, 37]. Nrf-2-deficient mice are more susceptible to oxidative stress [38] and inflammatory disorders including systemic inflammation [39], localized inflammation, and neuroinflammation [40]. Among the transcription factors regulating inflammation and oxidative stress, NF-kappaB is one of the most relevant factors in microglial cells [41]. Although the neurotoxic potential of BF has been reported, its impact on microglial cells remains unclear. To our knowledge, studies regarding BF-induced neuroinflammatory and oxidative stress in brain cells such as microglia are lacking. Thus, the aim of the present study was to assess the inflammatory and cytotoxic effects of BF in microglia as well as the neurotoxic effects in OHSCs.

## Methods

### Laboratory animals

The use of experimental animals was conducted with caution according to the regulations of the Ethics Committee of the University of Freiburg Medical Center and with the official approval from Regierungspräsidium Freiburg (Nr. X-13/06A). Animals were obtained from the Center of Experimental Models and Transgenic Services in Freiburg (CEMT-Freiburg). Generally, careful procedures and experimental setup were carried out in this work to minimize animal numbers and their suffering.

### Chemicals and reagents

An analytical standard of BF [2-methylbiphenyl-3-ylmethyl-(Z)-(1RS)-cis-3-(2-chloro-3,3,3-trifluoroprop-1-enyl)-2,2-dimethylcyclopropane carboxylate, 99.5%] was obtained from Sigma-Aldrich (Deissenhofen, Germany). BF was prepared as a stock solution of 100 mM dissolved in dimethyl sulfoxide (DMSO) and further diluted to appropriate concentrations in culture medium immediately prior to use [42]. 2′,7′-dichlorofluorescein diacetate (DCFH-DA) was obtained from Sigma-Aldrich (Deissenhofen, Germany). Lipopolysaccharide (LPS) from *Salmonella typhimurium* (Sigma-Aldrich, Deissenhofen, Germany) was resuspended in sterile phosphate-buffered saline (PBS) (5 mg/mL) as stock and subsequently used at a final concentration of 100 ng/mL. Clodronate disodium salt (Merck Chemicals, Darmstadt, Germany) was prepared as a stock solution of 1 mg/mL in ultrapure water and stored at − 20 °C. Propidium iodide (PI) dye was obtained from Life technologies (Darmstadt, Germany), prepared as a stock solution of 1 mg/mL in ultrapure water and stored at 4 °C. Dulbecco’s modified Eagle’s medium (DMEM), non-essential amino acids (NEAA), 4-(2-hydroxyethyl)-1-piperazineethanesulfonic acid (HEPES), Hank’s balanced salt solution (HBSS), and RPMI-1640 medium were purchased from Life Technologies. Penicillin, streptomycin, and 0.05% (*w*/*v*) trypsin/EDTA were obtained from Sigma-Aldrich. Fetal calf serum (FCS) was purchased from Bio&Sell (Feucht, Germany). Ethanol 100% (EthO) was obtained from Sigma-Aldrich (Deissenhofen, Germany). The CellTiter 96® AQueous One Solution Cell Proliferation Assay kit was obtained from Promega (Mannheim, Germany). EIAs for the determination of prostaglandin E_2_ (PGE_2_) were purchased from Cayman (distributed by Bertin Pharma, Montigny-le-Bretonneux, France) and ELISA kits for rat tumor necrosis factor-alpha (TNF-alpha) from eBioscience, Frankfurt, Germany.

### Primary glial cultures

Primary mixed glial cell cultures were established from the cerebral cortices of 1- to 3-day-old neonatal Sprague Dawley rats as previously described [43, 44]. Cerebral cortices were collected and meninges removed. Forebrains were then minced and gently dissociated by repeated pipetting in Dulbecco’s modified Eagle’s medium (DMEM) and filtered by passing through a 70-μm nylon cell strainer (BD Biosciences, Heidelberg, Germany). Cells were collected by centrifugation (1000×*g*, 10 min) and resuspended in DMEM (Life Technologies) containing 10% fetal calf serum (FCS) (Bio&Sell) and 1% penicillin and streptomycin antibiotics (40 and 40 μg/mL, respectively). Cells were then cultured on 10-cm cell culture dishes (Falcon, Heidelberg, Germany) with the density of 5 × 10^5^ cells/mL in 5% CO_2_ at 37 °C. After 12–14 days in vitro, floating microglia were harvested from mixed glia (astrocyte-microglia) cultures and re-seeded into cell culture plates at the density of 2 × 10^5^ cells/well. On the next day, the medium was removed to get rid of non-adherent cells and fresh medium was added. After 1 h, microglial cells were exposed to different concentrations of BF (1–20 μM) for 4 and 24 h; control cells were incubated with the solvent DMSO (0.1%).

### MTT assay and cell viability in primary microglia

Cell viability was measured by quantitative colorimetric assay with MTT-tetrazolio (MTT), as described previously [45]. Primary microglial cells (3 × 10^3^ cells/well) were seeded on 96-well cell culture plates and incubated for 24 h in 5% CO_2_ at 37 °C without treatment or treated with different concentrations of BF (0.1–100 μM). EthO 100% and DMSO 10% were used as positive controls to induce the cell death, thus, serving as positive controls (black columns). After treatment, cells were incubated with 20 μL of the CellTiter 96® AQueous One Solution Cell Proliferation Assay kit (Promega, Mannheim, Germany) for 4 h. Afterwards, supernatants were removed and cells were solubilized with DMSO to detect the intracellular formazan crystals formed in the viable cells. Finally, the absorbance of each well was measured at 490 nm by using an ELISA reader as described by the manufacturer.

### Lactate dehydrogenase measurement in primary microglia

Membrane integrity was evaluated by measuring lactate dehydrogenase (LDH) in the supernatants of untreated and treated cells. Primary microglial cells (3 × 10^3^ cells per well in 96-well plates) were incubated with different concentrations of BF (1–20 μM) for 4 and 24 h in 5% CO_2_ at 37 °C. Cell culture medium was collected and LDH activity was measured following manufacturer’s protocol (Biomagreb, ref. 20012 Ariana, Tunis, Tunisia). The percentage of LDH activity was determined as a percent of enzyme activity in the incubation medium to the total LDH activity. The reduction in absorbance due to NAD (H) oxidation was measured at 340 nm. The absence of the viable cells corresponds to 100% of LDH activity in the incubation medium.

### Measurement of reactive oxygen species contents in primary microglia

ROS levels in primary microglial cells were determined using 2,7-dichlorodihydrofluorescein (DCFH-DA). DCFH-DA enters cells passively and is deacetylated by esterase to non-fluorescent DCFH. DCFH reacts with ROS to form DCF, the fluorescent product. Primary microglial cells were seeded into 96-well culture plates (3 × 10^3^ cells/well) and treated with different concentrations of BF for 4 and 24 h. In brief, 5 μM H_2_DCF–DA >was added to each well. The fluorescence was measured every 1 h during a 4-h time period using a FL800-BioTek spectrofluorometer (Bio-Tek Instruments INC, Germany) with excitation/emission wavelengths of 488 nm/525 nm. ROS levels were expressed as the mean percentage of fluorescence absorbance in treated versus control cells.

### Determination of prostaglandin E_2_ and tumor necrosis factor alpha production in primary microglia

Primary microglial cells were left untreated or incubated with different concentrations of BF (1–20 μM) for 4 and 24 h. Afterward, supernatants were collected and then centrifuged at 1000×*g* for 5 min at 4 °C. EIA for PGE_2_ (Cayman, distributed by Bertin Pharma, Montigny-le-Bretonneux, France) and ELISA for TNF-alpha (eBioscience, Frankfurt, Germany) were performed according to the manufacturer’s instructions. For PGE_2_, standard concentrations of 39–2500 pg/mL were used and sensitivity of the assay was 36 pg/mL. For TNF-alpha, the standard concentrations were used in the interval of 16–2000 pg/mL and the detection limit was 16 pg/mL.

### Determination of nitric oxide production in primary microglia

Primary microglial cells (3 × 10^3^ cells/well) were pre-incubated with various concentrations of BF (1–20 μM) for 4 and 24 h. After the indicated time points, supernatants were collected and centrifuged at 10,000×*g* for 5 min. NO levels in the supernatants were determined using the Griess method [46]. One hundred microliters of supernatants were incubated with 100 μL of Griess reagent (Sigma-Aldrich, Deissenhofen, Germany) containing 1% sulfanilamide, 2% phosphoric acid, and 0.1% naphthyethylene diamide. After 20 min of incubation, absorbance was measured, at 540 nm. Nitrite concentrations in the cells were determined based on a sodium nitrite standard curve. Results were expressed as mean nitrite concentration (μM) ± SEM for each group.

### Measurement of hydrogen peroxide in primary microglia

Primary microglial cells (3 × 10^3^ cells/ well) were left untreated or treated with different concentrations of BF (1–20 μM) for 4 and 24 h in 5% CO_2_ at 37 °C. Afterwards, hydrogen peroxide (H_2_O_2_) formed by the microglial cells was determined by the ferrous ion oxidation-xylenol orange (FOX1) method [47]. The FOX1 reagent consists of 25 mM sulfuric acid, 250 μM ferrous ammonium sulfate, 100 μM xylenol orange, and 0.1 M sorbitol. Briefly, 100 μL of cell supernatants were added to 900 μL of FOX1 reagent, vortexed, and incubated for 30 min at room temperature. Solutions were then centrifuged at 12,000×*g* for 10 min. The amount of H_2_O_2_ in the supernatants was spectrophotometrically determined at 560 nm. Results were expressed as nmol per mg protein.

### Determination of lipid peroxidation in primary microglia

Primary microglial cells (3 × 10^3^ cells/ well) were left untreated or treated with different concentrations of BF (1–20 μM) and incubated for 4 and 24 h in 5% CO_2_ at 37 °C. The extent of lipid peroxidation in homogenates of primary microglial cells was then determined by measuring the release of a thiobarbituric acid reactive substance (TBARS) in terms of malondialdehyde (MDA) formation content and was measured using the thiobarbituric acid (TBA) colorimetric assay according to the Draper and Hadley (1990) method [48]. Briefly, cultures were washed with ice-cold PBS, pooled in 0.1 mol/L PBS/5% Triton X-100 buffered solution, and incubated for 1 h at 37 °C. Then, trichloroacetic acid (350 μl; 20% *w*/*v*) was added to 250 μL of cellular lysate and centrifuged (1000×*g* at 4 °C for 10 min). Aliquots (450 μL) of each supernatant were mixed with an equal volume of 0.5% (*w*/*v*) TBA. The mixture was boiled at 100 °C for 30 min. After cooling, MDA formation was measured at 520 nm and results were expressed as nmol of MDA/mg of protein.

### Measurements of antioxidant enzyme activities in primary microglia

Microglial cells (3 × 10^3^ cells/well) were plated and treated with different concentrations of BF (1–20 μM) for 4 and 24 h and then collected for the measurement of antioxidant enzyme activities. Catalase (CAT) activity was assayed by the decomposition of hydrogen peroxide according to the method of Aebi et al. [49]. A decrease in absorbance due to H_2_O_2_ degradation was monitored at 240 nm for 1 min, and the enzyme activity was expressed as nmol H_2_O_2_ consumed/min/mg protein. Superoxide dismutase (SOD) (MnSOD and Cu/ZnSOD) activities were evaluated by measuring the inhibition of pyrogallol activity as described by Marklund et al. [50]. This method is based on the competition between pyrogallol oxidation by superoxide radicals and superoxide dismutation by SOD. The specific Cu/Zn-SOD inhibition by potassium cyanide allows the Mn-SOD determination in the same conditions. Assays were monitored by spectrophotometry at 420 nm. One unit (U) corresponds to the enzyme activity required to inhibit half of pyrogallol oxidation. SOD activity was expressed as U/mg protein. Glutathione peroxidase (GPx) activity was measured according to Flohe and Gunzleret al. [51]. The enzyme activity was expressed as nmol of GSH oxidized/min/mg protein.

### Quantification of mRNA expression by real-time RT-PCR

Primary microglial cells were treated with BF (1–20 μM) for 4 and 24 h. Microglia RNA was then isolated using the Qiagen RNeasy Mini kit according to the manufacturer’s instructions (Qiagen, Germany), and cDNA was synthesized from 500 ng of total RNA using MMLV reverse transcriptase and random hexamers (Promega, Mannheim, Germany). The synthesized cDNA was used as template for the real-time qPCR amplification carried out by the CFX96 real-time PCR detection system using iQ™ SYBR supermix (LifeTechnologies, Darmstadt, Germany). Specific primer sequences were designed by using the Universal ProbeLibrary (Roche) and were obtained from Biomers (Ulm, Germany). These include primers for Nrf-2, NF-kappaBp65, TNF-alpha, COX-2, mPGES-1, and IL-6 (Table 1). Glyceraldehyde 3-phosphate dehydrogenase (GAPDH) served as an internal control for sample normalization, and the comparative cycle threshold Ct method was used for data quantification as described previously [52].

**Table 1:** Primer sequences for real-time PCR

Gene name	Primer sequences	
	Forward	Reverse
Nrf-2	5′-CAGCACATCCAGACAGACACCA-3′	5′CGTATTAAGACACTGTAACTCGGGAATGG-3′
NF-kBp65	5′-CTGTTTCCCCTCATCTTTCCCTC-3′	5′- TCCCGTGTAGCCATTGATCTTG-3′
COX-2	5′-ATG CTC TTCCGAGCTGTGCT-3′	5′- CATGGGAGTTGGGCAGTCAT-3′
mPGES-1	5′-ATGACTTCCCTGGGTTTGGTGATGGAG-3′	5′-TCACAGATGGTGGG CCACTTCCCAGA-3
TNF-alpha	5′-GTTCTATGGCCCAGACTGA-′3	5′-GTGGGTGAGGAGCACGTAGT-′3
IL-6	5′-AGTTGCCTTCTTGGGACTGA-′3	5′-TTCTGCAAGTGCATCATCGT-′3
GAPDH	5′-ACCACAGTCCATGCCATCAC-3′	5′-TCCACCACCCTGTTG CTGTA-3′

### Western blot analysis

Primary microglial cells were treated with BF and then incubated for 4 and 24 h with or without LPS (100 ng/mL), which was used as a positive control (black column). Cells were then washed with PBS and lysed in 1.3× sodium dodecyl sulfate (SDS) containing sample buffer without dithiothreitol (DTT). Protein concentrations were measured using the bicinchoninic acid (BCA) assay (Thermo Fischer Scientific, Waltham, MA). The optical density was read at 570 nm using a microplate reader, and concentrations were given based on the bovine serum albumin (BSA) protein standard curve. Immediately before electrophoresis, bromophenol blue and DTT (final concentration of 10 mM each) were added to the samples and 30 μg of protein from each sample were subjected to SDS-PAGE (polyacrylamide gel electrophoresis) on a 12% gel under reducing conditions. Proteins were then transferred onto a polyvinylidene fluoride (PVDF) membrane (Merck Chemicals, Schwalbach, Germany) by semi-dry blotting. The membrane was blocked for 1 or 2 h at room temperature using Rotiblock (Roth, Karlsruhe, Germany) for COX-2 or 5% blocking milk (BioRad, München, Germany) for the other proteins. Primary antibodies used were anti-COX-2 (1:500; Santa Cruz Biotechnology, Heidelberg, Germany), anti-mPGES-1 (1:6000; Agrisera, Vännas, Sweden), anti-Nrf-2 (1:1000; Santa Cruz Biotechnology), anti-beta-actin (1:5000, Sigma-Aldrich), and anti-NF-kappaB-p65 (1:1000; Santa Cruz Biotechnology). Primary antibodies were diluted in TBS-T and 1% BSA. Membranes were incubated with the primary antibody overnight at 4 °C, followed by extensive washing (three times for 15 min each in TBS containing 0.1% Tween 20). Proteins were detected by a 1-h incubation with horseradish peroxidase (HRP)-coupled rabbit anti-goat IgG (Santa Cruz, 1:100000), anti-Mouse IgG (GE Healthcare, 1:20000), or HRP-coupled donkey anti-rabbit (GE Healthcare, 1:25000) using chemiluminescence (ECL) reagents (GE Healthcare). All Western blot experiments were performed at least three times. The densitometric analysis of the Western blots was performed using Image J software 1.47v (National Institute of Health). The beta-actin signals served as normalization controls for the target proteins.

### Preparation of organotypic hippocampal slice cultures

Organotypic hippocampal slice cultures (OHSCs) were prepared as described earlier [43, 44, 53] with minor modifications. In brief, slice cultures were prepared from 1- to 3-day-old C57BL/6 wild-type mice under sterile conditions. After decapitation, brains were removed and the hippocampi from both hemispheres were acutely isolated in ice-cold serum-free Hank’s balanced salt solution (HBSS), supplemented with 0.5% glucose (Sigma-Aldrich) and 15 mM HEPES. Isolated hippocampi were cut into 350–375-μM-thick slices using a tissue chopper (McIlwain) and were transferred to 0.4 μM culture plate inserts (Merck Chemicals, PICM03050). The inserts containing four to six slices were placed in six-well plates containing 1.2 mL of culture medium per well. Culture medium (pH 7.2) consisted of 0.5× minimum essential medium (MEM) containing 25% heat-inactivated horse serum, 25% BME basal medium without glutamate, 2 mM glutamax (all from LifeTechnologies), and 0.65% glucose (Sigma-Aldrich). The slice cultures were kept at 37 °C in a humidified atmosphere (5% CO_2_), and the culture medium was refreshed the first day after preparation and every consecutive 2 days.

### Depletion of microglia from slice cultures

To deplete microglia from OHSCs, slice cultures were placed immediately after preparation either on culture medium containing clodronate encapsulated in liposomes (Chlodronate Liposomes, Haarlem, Netherlands) or culture medium containing 100 μg/mL clodronate disodium salt (Merck Chemicals, Darmstadt, Germany) [43, 44]. Twenty-four hours after preparation, the slice cultures were washed briefly with prewarmed PBS and reincubated in fresh culture medium for 7 days before use. The OHSC culture medium was changed every second day.

### Immunostaining of organotypic hippocampal cultures

For immunohistochemical analysis, controls and BF-challenged slice cultures were shortly rinsed in PBS and fixed with 4% paraformaldehyde (PFA) overnight at 4 °C. After fixation, the slice cultures were rinsed in PBS and pre-incubated with 5% normal goat serum (NGS, Vector) in PBS containing 0.3% Triton X-100 (PBS+) overnight. Subsequently, the slice cultures were incubated with the appropriate primary antibodies overnight in 1% NGS/ PBS+ at 4 °C. The following primary antibodies were used: rabbit-anti-Iba-1 (1:1000, Wako 019-19741) for detection of microglia, mouse-anti-GFAP (1:600, Chemicon MAB3402) for detection of astrocytes, and mouse-anti-NeuN (1:1000, Chemicon MAB377) for detection of neuronal nuclei. The secondary antibodies used were a donkey-anti-mouse-Alexa488 (Molecular Probes) for NeuN, donkey-anti-rabbit-Alexa633 (Molecular Probes) for Iba-1, and goat-anti-mouse-Cy3 (Jackson IR Laboratories) for GFAP. Imaging was carried out using laser scanning ZEISS LSM 510 META microscope. To quantify neuronal cell death in response to BF-induced neurotoxicity, slice cultures were incubated with 2 μg/mL PI during and after the BF challenge [44, 54]. Immunofluorescently stained OHSCs were analyzed by confocal laser scanning microscopy using a ZEISS LSM 510 META upright microscope (objective, C-apochromat × 10), and the number of PI-positive cells were quantified using ImageJ software (as described in [54]). The percentage of cell death was determined by calculation of PI area fraction as a percentage of NeuN area fraction. Quantifications of Iba-1 and GFAP area fractions after BF treatment were calculated as a percentage of untreated control area fraction using ImageJ software [54, 55].

### Statistical analysis

All statistical analyses were carried out using GraphPad Prism 6.0 for Windows (GraphPad Software, San Diego, CA). Results were expressed as mean ± standard errors (mean ± SEM). Multiple comparisons data were analyzed using one-way ANOVA followed by post hoc Student–Newman–Keuls test. Two-way ANOVA with Bonferroni post hoc test was used for grouped analyses. For each experiment, a *p* value < 0.05 was considered statistically significant.

## Results

### BF-induced cytotoxicity in primary microglia

**Fig. 1:**
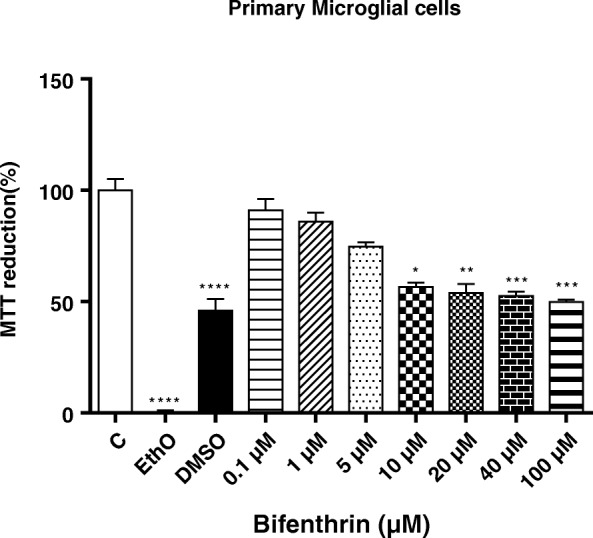
Effects of BF on cell viability in primary microglia cells. Microglial cells were exposed to different concentrations of BF (0.1–100 μM) for 24 h. Cell viability was measured using the MTT assay, and data were normalized as percentage of negative control with DMSO (0.1%) (C, white column). EthO 100% and DMSO 10% were used as positive controls to induce cell death (black columns). Statistical analyses were carried out by using one-way ANOVA followed by post hoc Student–Newman–Keuls test. Results are expressed as means ± SEM from three independent experiments. *p < 0.05; **p < 0.01; **p < 0.001 compared with untreated control (DMSO, white column)

The cytotoxicity of BF on primary microglia was assessed using MTT assay (Fig. 1). Microglial cells were left untreated or exposed to various concentrations (0.1–100 μM) of BF for 24 h. Furthermore, ethanol (100%) and DMSO (10%) were used as positive controls to induce cell death. Exposure to high concentrations (10–100 μM) of BF for 24 h induced a significant decrease in the cell viability with maximal effects at 100 μM (*p* < 0.05) (Fig. 1). In contrast, no significant cell death was observed at lower concentrations (0.1, 1, and 5 μM) of BF (Fig. 1). The positive control ethanol induced 100% cell death and DMSO approximately 55% (Fig. 1, black columns).

### Effects of BF on lactate dehydrogenase and reactive oxygen species formation in primary microglia

**Fig. 2:**
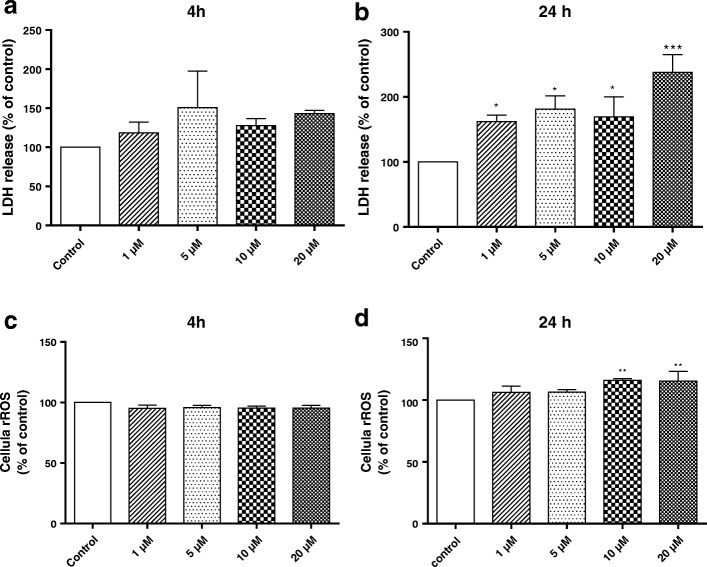
Effects of BF on lactate dehydrogenase and reactive oxygen species formation in primary microglia. Microglial cells were exposed to different concentrations of BF (1–20 μM) for 4 and 24 h. Release of LDH (**a**, **b**) and formation of ROS (**c**, **d**) were measured by a spectrofluorometer. Statistical analyses were carried out by using post hoc Student–Newman–Keuls test. Results are expressed as means ± SEM of three independent experiments. *p < 0.05; **p < 0.01; **p < 0.001 compared with control (DMSO, white column)

Primary microglial cells were chosen to investigate the effect of BF on oxidative stress and inflammatory mediators. In a first step, microglial cells were treated with different concentrations of BF (Fig. 2a–d). Treatment for 4 h with BF (1–20 μM) did not affect LDH activity and ROS production (Fig. 2a, c). In contrast, a significant increase of LDH and ROS production was observed 24 h after BF treatments at concentrations of 1–20 μM and 10–20 μM, respectively (Fig. 2b, d).

### Effects of BF on prostaglandin E_2_, nitric oxide, and tumor necrosis factor-alpha production in primary microglia

**Fig. 3:**
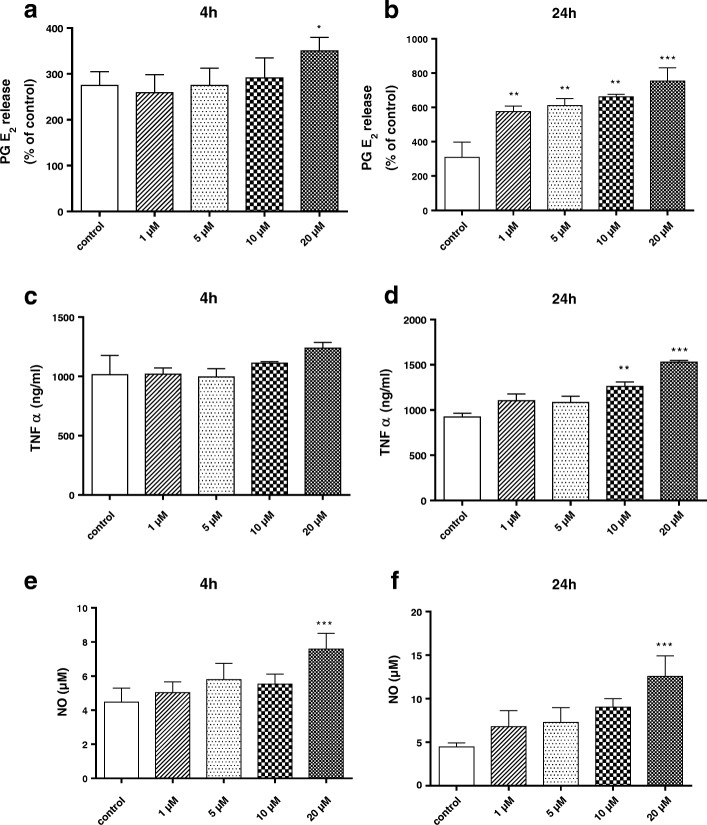
Effects of BF on prostaglandin E_2_, nitric oxide, and tumor necrosis factor alpha production in primary microglia. Microglial cells were exposed to different concentrations of BF (1–20 μM) for 4 and 24 h. At the end of incubation, cell supernatants were collected and the production of PGE_2_ (**a**, **b**), TNF-alpha (**c**, **d**), and NO (**e**, **f**) was determined. Statistical analyses were carried out by using one-way ANOVA followed by post hoc Student–Newman–Keuls test. Results are expressed as means ± SEM of three independent experiments. *p < 0.05; **p < 0.01; **p < 0.001 compared with control (DMSO, white column)

To further investigate the direct effects of BF on neuroinflammatory mediators, primary microglial cells were exposed to different concentrations of BF (1, 5, 10, and 20 μM) for 4 and 24 h and levels of PGE_2_, NO, and TNF-alpha were determined in the cell supernatants (Fig. 3). Our data showed that incubation with BF for 24 h induced a marked increase in PGE_2_, NO, and TNF-alpha production as compared to the vehicle-treated controls, especially at the high concentrations (10–20 μM) (Fig. 3b, d, and f). However, 4 h after BF treatment, only the highest concentration (20 μM) induced PGE_2_ release and increased NO levels, while TNF-alpha production was not affected (Fig. 3a, c, and e).

### Effects of BF on oxidative stress markers in primary microglia

**Fig. 4:**
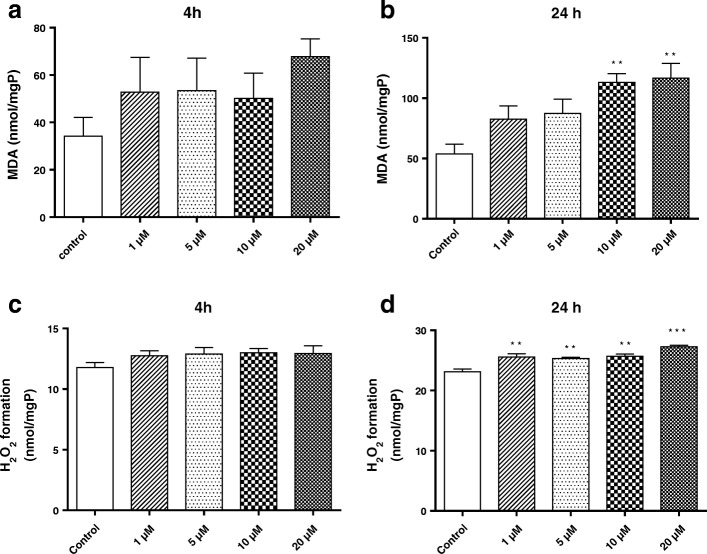
Effects of BF on oxidative stress markers in primary microglia. Microglial cells were exposed to different concentrations of BF (1–20 μM) for 4 and 24 h. Lipid peroxidation (MDA) (**a**, **b**) and hydrogen peroxides (H_2_O_2_) (**c**, **d**) levels were measured by a spectrometer. Statistical analyses were carried out by using one-way ANOVA followed by post hoc Student–Newman–Keuls test. Results are expressed as means ± SEM of three independent experiments. *p < 0.05; **p < 0.01; **p < 0.001 compared with control (DMSO, white column)

We next investigated the effects of BF on two common oxidative stress markers MDA and H_2_O_2_. Our results demonstrate that 24 h of BF treatment significantly increased the levels of MDA (at 10–20 μM) and H_2_O_2_ (at 1–20 μM) (Fig. 4b, d). No significant effects were observed following 4 h of BF treatment on MDA and H_2_O_2_ levels (Fig. 4a, c).

### Effects of BF on enzymatic markers in primary microglia

**Fig. 5:**
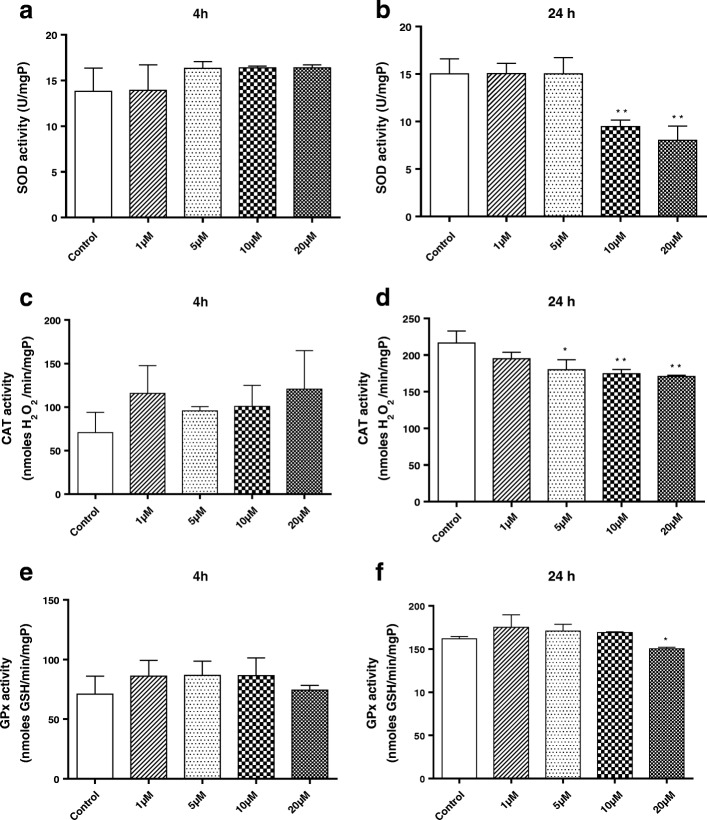
Effects of BF on enzymatic markers of oxidative stress in primary microglia. Microglial cells were exposed to different concentrations of BF (1–20 μM) for 4 and 24 h. Enzyme activities of superoxide dismutase (SOD) (**a**, **b**), catalase (CAT) (**c**, **d**), and glutathione peroxidase (GPx) (**e**, **f**) in primary microglial cells were spectrometrically determined. Statistical analyses were carried out by using one-way ANOVA followed by post hoc Student–Newman–Keuls test. Results are expressed as means ± SEM of three independent experiments. *p < 0.05; **p < 0.01; **p < 0.001 compared with control (DMSO, white column)

Intracellular levels of antioxidant enzymes, including SOD, CAT, and GPx, were investigated in primary microglial cells incubated with various concentrations of BF (1, 5, 10, and 20 μM) at 4 and 24 h (Fig. 5a–f). Our data demonstrated a decrease in SOD activity only with the high concentrations of BF (10 and 20 μM) at 24 h (Fig. 5b). GPx activity was significantly decreased only with the highest concentration of BF (20 μM) at 24 h (Fig. 5f). Furthermore, a marked decrease in the activity of CAT by BF was observed at different concentrations (5, 10, and 20 μM) after 24 h of treatment (Fig. 5d). In contrast, no significant decrease in the activities of endogenous antioxidant enzymes (SOD, CAT, and GPx) was observed after 4 h of BF treatment (Fig. 5a, c, and e).

### BF exposure increases the expression of pro-inflammatory markers in primary microglia.

**Fig. 6:**
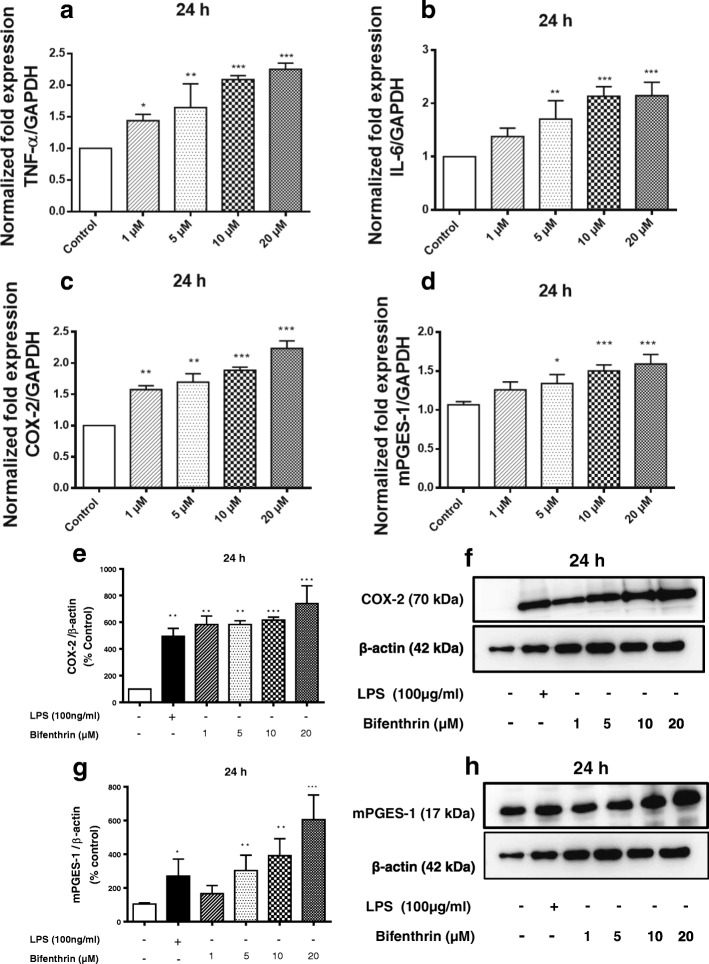
BF exposure increases the expression of pro-inflammatory markers in primary microglia cells. Microglial cells were exposed to different concentrations of BF (1–20 μM) for 24 h. Gene expression of TNF-alpha (**a**), IL-6 (**b**), COX-2 (**c**), and mPGES-1 (**d**) was analyzed by real-time quantitative PCR. GAPDH was used as an internal control for normalization, and data were quantified by using the comparative cycle threshold Ct method. Similarly, cells were treated with BF and thereafter incubated with or without LPS (100 ng/mL) as a positive control (black column) for 24 h. Whole cell lysates were subjected to Western blot for COX-2 (**e**, **f**), mPGES-1 (**g**, **h**), and beta-actin. To confirm equal sample loading, beta-actin was used for normalization. Data are presented as percentage control of DMSO. Statistical analyses were carried out by using one-way ANOVA followed by post hoc Student–Newman–Keuls test. Results are expressed as means ± SEM of three independent experiments. *p < 0.05; **p < 0.01; **p < 0.001 compared with control (DMSO, white column)

It was further examined whether BF exposure increased mRNA expression of pro-inflammatory cytokines in primary microglia. Treatment with BF (1–20 μM) for 4 and 24 h increased IL-6 and TNF-alpha mRNA expression in a concentration-dependent manner (Fig. 6a, b; Additional file 1 A1-A and B). In addition, BF mediated a significant concentration-dependent increase of COX-2 and mPGES-1 at the mRNA and protein levels at 24 h (Fig. 6c–h). After 4 h of incubation with BF, a marked increase in mRNA and protein levels of COX-2 was observed starting from 5 μM (see Additional file 1 A1-C, E, and F). Although a significant increase of mPGES-1 at the mRNA and protein levels was observed with 20 μM BF at 4 h, lower concentrations of BF (1–10 μM) did not exert any effects on this enzyme at the same time point (Additional file 1).

### BF exposure increases the expression of Nrf-2 and NF-kappaB in primary microglia

**Fig. 7:**
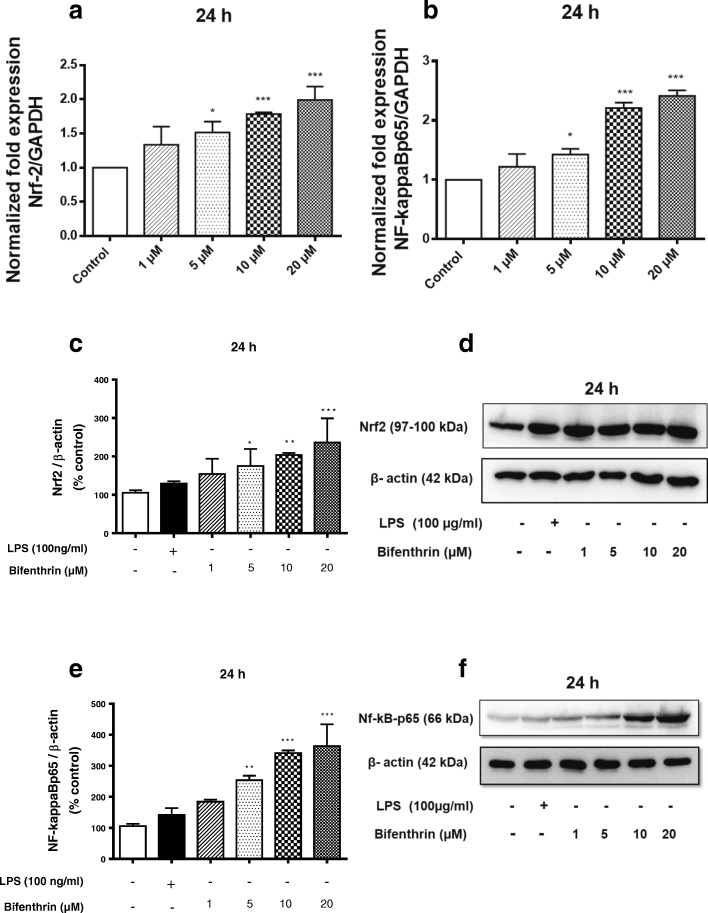
BF exposure increases the expression of Nrf-2 and NF-kappaB in primary microglia. Microglial cells were exposed to different concentrations of BF (1–20 μM) for 24 h. Gene expression of Nrf-2 (**a**) and NF-kappaBp65 (**b**) was analyzed by real-time quantitative PCR. GAPDH was used as an internal control for normalization, and data were quantified by using the comparative cycle threshold Ct method. Similarly, cells were treated with BF and thereafter incubated with or without LPS (100 ng/mL) as a positive control (black column) for 24 h. Whole cell lysates were subjected to Western blot for Nrf-2 (**c**, **d**), NF-kappaBp65 (**e**, **f**), and beta-actin. Data are presented as percentage control of DMSO. Statistical analyses were carried out by using one-way ANOVA followed by post hoc Student–Newman–Keuls test. Results are expressed as means ± SEM of three independent experiments. *p < 0.05; **p < 0.01; **p < 0.001 compared with control (DMSO, white column)

Inflammation and oxidative stress are controlled by several cellular aspects; among them, Nrf-2 and NF-kappaB are the two key transcription factors with a major function in these processes [56]. Therefore, we evaluated the effects of BF on Nrf-2 and NF-kappaB pathways by qPCR and Western blot analysis. We observed a significant increase in mRNA expression of Nrf-2 and NF-kappaBp65 after 24 h of incubation with BF in primary microglia (Fig. 7a, b), whereas at 4 h, only NF-kappaBp65 expression was increased (Additional file 2). Increased mRNA expression was detectable starting at 5 μM and reached its maximum at 20 μM of BF.

Four hours of incubation with BF had no significant effect on the protein level of Nrf-2 (Additional file 2), whereas NF-kappaBp65 protein level was significantly increased by BF starting at 5 μM and maximal levels were identified at 20 μM (Additional file 2). Similar to the results obtained from mRNA analysis, Western blot data of the 24-h experiment showed a dose-dependent increase of Nrf-2 and NF-kappaBp65 proteins starting at 5 μM of BF (Fig. 7c–f). The highest levels of Nrf-2 and NF-kappaBp65 protein levels were observed at 20 μM of BF.

### BF-induced neurodegeneration in mouse organotypic hippocampal slice cultures

**Fig. 8:**
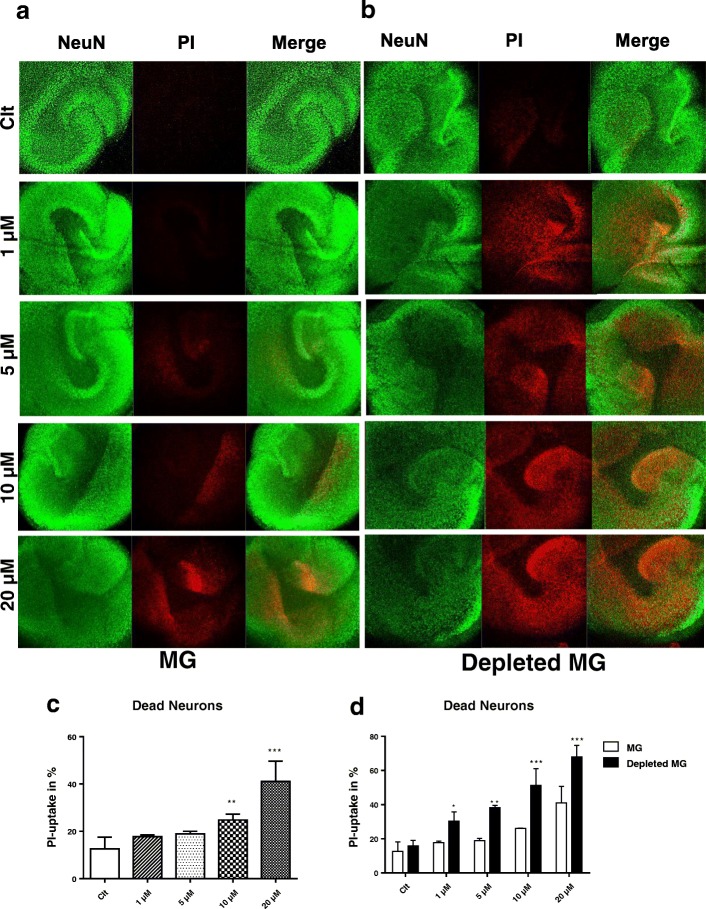
BF-induced neurodegeneration in mouse organotypic hippocampal slice cultures. After 7 days of incubation in culture, OHSCs were left untreated (control) or treated with different concentrations of BF (1–20 μM). Immunolabelling for neuron (NeuN/PI) treatment with BF in microglia-depleted (**b**–**d**) and microglia-containing OHSCs (**a**, **c**, and **d**), PI (red) is co-localized with the neuronal nuclear marker NeuN (green) (C, white arrow). Data are mean ± SEM from two independent experiments with six slice cultures/well for each condition. Statistical analyses were carried out by one-way ANOVA followed by post hoc Student–Newman–Keuls test between OHSC treated with different concentrations of BF (1–20 μM) and OHSC control group (**c**), as well as by two-way ANOVA with Bonferroni post hoc test between OHSCs depleted and non-depleted microglia with different concentrations of BF (1–20 μM) (grouped analysis) (**d**). Statistical significance is denoted by *p < .05, **p < .01, ***p < .001

To examine possible neurocytotoxic effects of BF ex vivo, we treated OHSCs for 24 h with BF (1–20 μM) and performed PI and NeuN immunohistochemistry. Confocal images showed that BF treatment at higher concentrations (10 and 20 μM) induced neuronal death (colocalization of PI with NeuN) in all hippocampus layers (CA1, CA3, and DG) with 12% (10 μM) and 31% (20 μM) of PI uptake. However, BF concentrations lower than 10 μM did not induce significant cell death in any of the neuronal regions (Fig. 8a, c, and d).

### Depletion of microglia enhances neuronal death mediated by BF

We next aimed to examine whether microglial depletion in OHSCs might affect BF-induced neuronal cell death. We eliminated microglia by clodronate liposomes and subsequently examined neuronal cell death. We recently showed that clodronate treatment successfully depletes microglia without affecting other OHSC cells [44].

OHSCs with and without microglia were treated with different concentrations (1–20 μM) of BF for 24 h. Following microglial depletion, severe neuronal death was observed with all BF concentrations (1–20 μM) as compared to untreated controls and microglia-containing OHSCs (Fig. 8b–d). In microglia-depleted OHSCs, BF induces neurotoxicity even at the low concentrations of 1 μM with 13% and of 5 μM with 20% differences in increased cell death between microglia-depleted and microglia-containing OHSCs, respectively. Cytotoxic effects of BF were severe and further increased at the high concentrations of 10 and 20 μM BF in the microglia-depleted OHSCs compared to microglia-containing OHSCs (Fig. 8a–d).

### BF treatment does not induce cell death of microglia and astrocytes in OHSCs

**Fig. 9:**
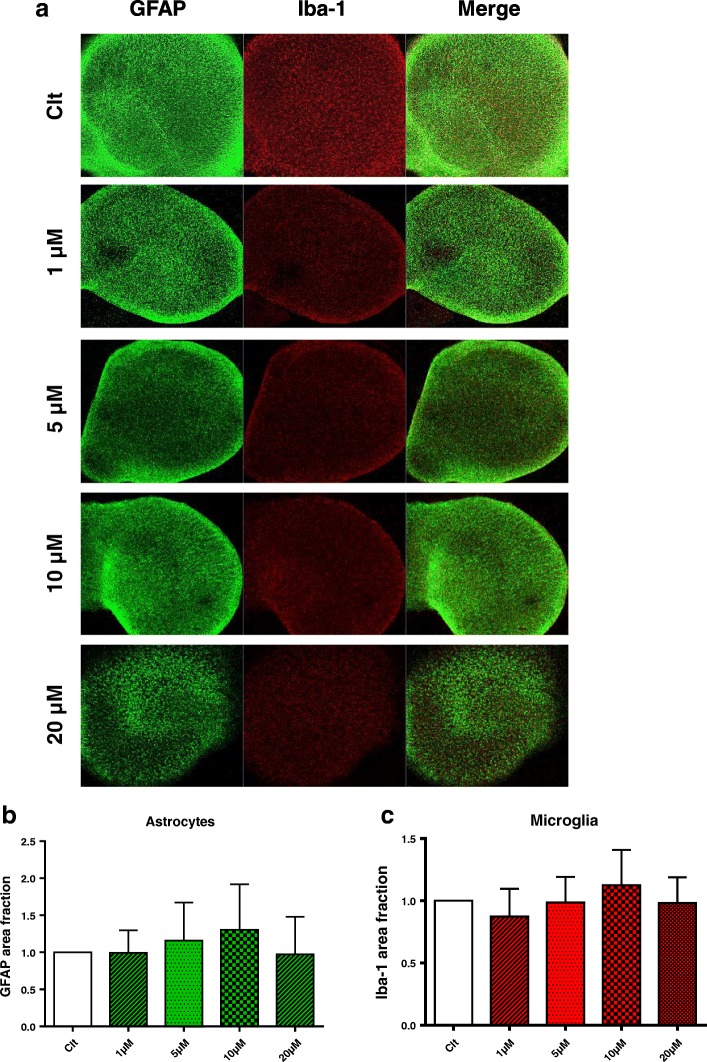
BF treatment does not cause cell death of microglia and astrocytes in OHSCs. **a**, **b** Immunolabelling for astrocytes were stained with anti-GFAP (GFAP in green) at 24 h following BF exposure; **a**, **c** microglial cells were fixed at 24 h following BF exposure and stained with anti-Iba-1 (Iba-1 in red). Data are mean ± SEM from two independent experiments with six slice cultures/well for each condition

Confocal images of Iba-1 staining showed no visible differences between BF treatments and controls in microglia (Fig. 9a). In addition, images of the astrocytic marker GFAP demonstrated no changes between BF treatments and controls (Fig. 9a). Quantifications of GFAP and Iba-1-positive staining area confirmed that BF does not induce microglial or astrocytic cell death in OHSCs (Fig. 9b, c).

## Discussion

The effects of pesticides on the brain and the accompanied cellular responses related to these effects are poorly understood [57–60]. Although the toxic responses of pyrethroids in various cell types have been documented and neurotoxic effects of BF are well known, their effects on microglial activation in vitro and neuronal cell death in OHSC ex vivo are not investigated so far in detail [11, 23, 61].

The main objective of the present study was to characterize the modulatory effects of BF on microglia-mediated neuroinflammation and neuronal death as a possible consequence and to investigate the neurotoxic impact of BF in OHSCs in the presence or absence of microglia, showing that microglia exert protective functions against BF toxicity. To our knowledge, this is the first study to show cytotoxic effects of BF in microglial cells and in OHSC slice cultures in order to describe possible pathways involved in the oxidative and inflammatory responses related to BF exposure.

We first examined the hypothesis that BF might induce microglial cytotoxicity. We showed here that BF at concentrations of 10 μM or higher caused cytotoxicity, as shown by decreased cell viability levels and increased LDH release. In a similar investigation, it has been shown that DM (type II SP) exposure induces cytotoxicity in SH-SY5Y cells [62]. It has been shown that activated microglia secrete a variety of factors, including ROS, nitrogen species, cytokines, prostaglandins, and chemokines, which can subsequently results in tissue injury or promote neurotoxicity [63–67]. Furthermore, excessive ROS have serious deleterious effects on cell components, such as lipids, proteins, and DNA, and lead to oxidative stress [68, 69]. In this study, we demonstrated that BF treatment significantly augments ROS production in primary microglia at different time points. This result was consistent with previous studies on enantioselective oxidative damage to human Hep G2 cells and amnion epithelial cells by BF [70].

Oxidative stress refers to the imbalance between the concentrations of ROS and the antioxidative defense mechanisms of the body [71]. However, many studies showed that pesticides induce oxidative damage and alter the defense system of detoxification and scavenging enzymes [72]. These xenobiotics impair the cellular function and enzyme activity and produce cytotoxic changes through the generation of free radicals. In the present study, BF treatment resulted in a significant decrease in SOD, CAT, and GPx activities in primary microglia, suggesting a BF-induced dysfunction of the antioxidant defense system and confirming the increased production of free radicals caused by the treatment with BF. These findings are similar to those described by El-Demerdash et al. [73], who revealed decreased SOD, CAT, and GPx activities in rat brain exposed to organophosphate and pyrethroid insecticides. Changes in these oxidative stress biomarkers have been reported to be an indicator of the tissue’s ability to cope with oxidative stress. Additionally, oxidative stress might induce significant lipid and protein peroxidation in cells. As one part of TBARS, MDA was regarded as a marker of lipid oxidation and was the most abundant individual aldehyde resulting from lipid peroxidation [74]. The present study revealed a significant increase in MDA, NO, and H_2_O_2_ levels in BF-treated primary microglia. In similar settings, it was reported that the pesticide PM induced enantioselective oxidative stress in rat PC12 cells in a dose-dependent manner [75]. This result is consistent with our previous studies in which lambda-cyhalothrin induced oxidative stress and ultimately neuronal damage in rat hippocampus [12].

To counter oxidative stress, microglia, along with other cells of the central nervous system, produce endogenous antioxidants that are regulated predominately through various tightly regulated defense mechanisms to maintain the ROS balance in tissue cells [76–78]. Nrf-2 is a transcription factor that acts as a key regulator of antioxidant-responsive gene such as heme oxygenase 1 (HO-1) and quinone oxidoreductase 1 (NQO1) in tissues and cells and plays a role in acute cerebral lesions and in neurodegenerative disorders [79–81]. Excessive intercellular ROS contribute to Nrf-2 activation to exert protective functions through inducing its target antioxidant genes [82]. In turn, Nrf-2 defense system activation relieves oxidative stress damage by modulating ROS and inflammatory processes [83]. In addition, previous studies have demonstrated that DM, a prototype of widely used pyrethroid pesticides, activates the Nrf-2 pathway in PC12 rat cells [84]. In the current study, we were able to show that 24 h of exposure to BF increased Nrf-2 expression and synthesis in primary microglial cells. This finding is particularly important because the progression of cognitive decline and neurodegenerative pathologies is closely associated with increased oxidative stress and microglial activation [68, 85]. These results are also in line with previous studies [19, 86], in which a relationship between AA, DM, and PM exposure in vitro and found increased cellular and nuclear accumulation of Nrf-2 was established, which in turn activated the expression of Nrf-2-regulated oxidative stress response genes. This suggests that the capacity of protection against oxidative damage offered by the Nrf-2-triggered antioxidant proteins may be limited and the system was overwhelmed by the BF exposure.

On the other hand, the neuroinflammatory response during most chronic neurodegenerative diseases is mediated by the activation of glial cells especially primarily microglia [87, 88, 89, 90]. Activated microglial cells produce several inflammatory cytokines such as TNF-alpha, IL-1beta, and IL-6, which are critical in regulating the immune responses in the CNS [55, 91, 92]. However, chronic activation of microglial cells excessively prolongs the inflammatory response and causes neuronal damage, which accelerates the development of neurodegenerative diseases [93, 94]. This response involves the additional production of inflammatory molecules, reactive oxygen species, and other mediators that may lead to detrimental effects [95–97]. NF-kappaB plays a pivotal role in the regulation of the expression of COX-2 and inflammatory cytokines such as TNF-alpha [98, 99]. Our current study demonstrated that BF remarkably increases the expression of NF-kappaB, COX-2, IL-6, and TNF-alpha in microglia, which is associated with increased NO, TNF-alpha, and PGE_2_ levels in primary microglial cells. Our findings are in agreement with those reported by Zhao et al. [19], who demonstrated that AA significantly increased NF-kappaB and proinflammatory mediators such as IL-1beta, IL-6, and TNF-alpha in astrocytes and microglial cells.

As described in our previous studies, the mPGES-1 and COX-2 enzyme pathway plays a significant role in microglia regulation. mPGES-1 is the terminal enzyme for the biosynthesis of PGE_2_ during inflammation and is normally functionally coupled with COX-2 with few exceptions [100, 101]. The binding of ROS to COX-2 directly elicits its activation, causing overexpression of COX-2 [102]. Our findings demonstrated an increase of COX-2 and mPGES-1 after BF incubation at both mRNA and protein levels.

**Fig. 10:**
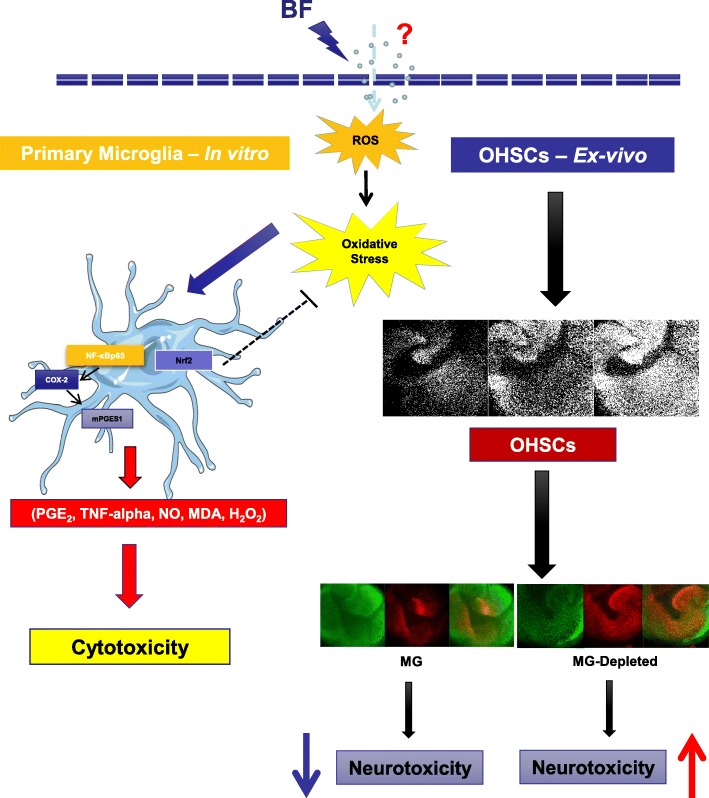
Schematic representation of BF cytotoxic effects in primary microglia and organotypic hippocampal slice cultures (OHSCs). Effects of exposure to BF in primary microglia on the production of reactive oxygen species (ROS), release of oxidative stress markers (lipid peroxidation (MDA), nitric oxide (NO), hydrogen peroxides (H_2_O_2_) and the inflammatory mediators cyclooxygenase-2 (COX-2), microsomal prostaglandin synthase-1 (mPGES-1), prostaglandin E2 (PGE_2_), and tumor necrosis factor-alpha (TNF-alpha)). Effects of BF on the activation of both nuclear erythroid-2 like factor-2 (Nrf-2) and nuclear factor kappaB (NF-kappaB). (B) Effect of BF treatment on organotypic hippocampal slice cultures (OHSCs - Ex-Vivo) in the presence or absence of microglia (MG) in slice cultures

Wardyn et al. [56] provided compelling evidence that Nrf-2 and NF-kappaB orchestrate the balance of cellular redox status and responses to stress and inflammation. Most importantly, environmental toxin-induced neurotoxicity might be mediated by induction of antioxidant genes through activation of NF-kappaB and Nrf-2 [19, 103–105]. Several lines of evidence suggest that Nrf-2 regulates the inflammatory response by inhibiting pro-inflammatory NF-kappaB activation through maintenance of redox homeostasis. The activation of the NF-kappaB signaling pathway seems to be responsive to excessive ROS generation, thus affecting the redox-sensitive NF-kappaB signaling pathway [106]. However, many pathological and/or physiological stimuli often activate both NF-kappaB and Nrf-2 signaling [107]. Indeed, when PC12 cells are treated for 48 h with paraquat, a widely used non-selective herbicide, a marked cellular toxic effect accompanied by a sustained ROS generation and NO production was observed [108]. Under these experimental conditions, nuclear protein levels of Nrf-2 and NF-kappaB were both increased, with maximal translocation of NF-kappaB. Our findings suggest that Nrf-2 and NF-kappaB play crucial roles in BF-induced oxidative stress and are further associated with neurotoxicity in primary microglia cells (Fig. 10).

Despite increasing evidence for severe neurotoxic effects of BF, few studies focused on neuronal cell models, whereby microglial cells have been mostly neglected. Immunohistochemical analyses of organotypic hippocampal slice cultures (OHSCs) treated with BF revealed the induction of neuronal cell death. OHSCs are known to preserve many morphological and functional features of the in vivo environment over long periods in culture and have been used in many models of excitotoxicity [43, 54, 109, 110]. In our current study, BF treatment of OHSCs resulted in a significant concentration-dependent neuronal cell death in all neuronal layers. Comparable neuronal damage in OHSCs is observed by kainic acid (KA) and N-Methyl-D-aspartic acid (NMDA) [43, 110]. Excitingly, upon microglial depletion, BF induces neurotoxicity even in low concentrations of 1 and 5 μM, which were not affected in the presence of microglia. Neurotoxic effects of BF were strongly exaggerated using high concentrations of BF (10 and 20 μM) inducing severe neuronal death if compared to OHSCs containing microglia. This data suggest a neuroprotective function for microglia against BF cytotoxicity. In this context, future investigations should unravel the underlying mechanism beyond this microglia protective effect in OHSCs. This result was consistent with the previous report by Vinet et al. [31], who demonstrated that depletion of microglia resulted in severe neuronal loss following NMDA excitotoxicity. We were able to observe that BF treatment affects neither microglial cells nor astrocyte populations in OHSCs, indicating specific neuronal vulnerability towards BF.

## Conclusion

In conclusion, our findings identified BF as a potentiator of oxidative stress and inflammatory mediators in primary microglial cells that ultimately contributed to cytotoxicity. Furthermore, we provided evidence that BF promotes neuronal death in OHSCs and found that microglia exert protective function against BF neurotoxicity.

## Additional files

Additional file 1: BF exposure increases the expression of pro-inflammatory markers in primary microglia cells. Microglial cells were exposed to different concentrations of BF for 4 h. Gene expression of TNF-alpha (A), IL-6 (B), COX-2 (C), and mPGES-1 (D) was analyzed by real-time quantitative PCR. GAPDH was used as an internal control for normalization, and data were quantified by using the comparative cycle threshold Ct method. Similarly, cells were treated with BF and thereafter incubated with or without LPS (100 ng/mL) as a positive control (black column) for 4 h. Whole cell lysates were subjected to Western blot for COX-2 (E and F), mPGES-1 (G and H), and beta-actin. To confirm equal sample loading, beta-actin was used for normalization. Moreover, data are presented as percentage control of DMSO. Statistical analyses were carried out by using one-way ANOVA followed by post hoc Student–Newman–Keuls test. Results are expressed as means ± SEM of three independent experiments. *p < 0.05; **p < 0.01; **p < 0.001 compared with control (DMSO, white column). (PDF 212 kb)

Additional file 2: BF exposure increases the expression of Nrf-2 and NF-kappaB in primary microglia. Microglial cells were exposed to different concentrations of BF (1–20 μM) for 4 h. Gene expression of Nrf-2 (A) and NF-kappaBp65 (B) was analyzed by real-time quantitative PCR. GAPDH was used as an internal control for normalization, and data were quantified by using the comparative cycle threshold Ct method. Similarly, cells were treated with BF thereafter incubated with or without LPS (100 ng/mL) as a positive control (black column) for 4 h. Whole cell lysates were subjected to Western blot for Nrf-2 (C and D), NF-kappaBp65 (E and F), and beta-actin. Data are presented as percentage control of DMSO. Statistical analyses were carried out by using one-way ANOVA followed by post hoc Student–Newman–Keuls test. Results are expressed as means ± SEM of three independent experiments. *p < 0.05; **p < 0.01; **p < 0.001 compared with control (DMSO, white column). (PDF 258 kb)

## Abbreviations

AA: Acrylamide
AB: Antibody
AD: Alzheimer’s disease
AL: Allethrin
BCA: Bicinchoninic acid
BF: Bifenthrin
BME: Basal Medium Eagle
BSA: Bovine serum albumin
CAT: Catalase
CM: Cypermethrin
CNS: Central nervous system
COX: Cyclooxygenase
DCFH-DA: 2′,7′-dichlorofluorescein diacetate
DM: Deltamethrin
DMEM: Dulbecco’s modified Eagle’s medium
DMSO: Dimethyl sulfoxide
DPBS: Dulbecco’s phosphate-buffered saline
DTT: Dithiothreitol
ECL: Chemiluminescence
EthO: Ethanol
FBS: Fetal bovine serum
FCS: Fetal calf serum
FOX1: Ferrous ion oxidation-xylenol orange
GAPDH: Glyceraldehyde 3-phosphate dehydrogenase
GFAP: Glial fibrillary acidic protein
GPx: Glutathione peroxidase
H_2_O_2_: Hydrogen peroxide
HBSS: Hank’s balanced salt solution
HEPES: 4-(2-hydroxyethyl)-1-piperazineethanesulfonic acid
HRP: Horseradish peroxidase
Iba-1: Ionized calcium-binding adapter molecule 1
IL: Interleukin
iNOS: Nitric oxide synthase
LDH: Lactate dehydrogenase
LPS: Lipopolysaccharide
MDA: Malondialdehyde
MEM: Minimum Essential Medium
mPGES: Microsomal prostaglandin E synthase
MS: Multiple sclerosis
MTT: MTT-tetrazolio
NEAA: Non-essential amino acids
NF-kappaB: Nuclear factor kappa-light-chain-enhancer of activated B cells
NGS: Normal goat serum
NMDA: N-Methyl-D-aspartic acid
Nrf-2: Nuclear erythroid-2 like factor-2
OHSCs: Organotypic hippocampal slice cultures
PBS: Phosphate-buffered saline
PFA: Paraformaldehyde
PG: Prostaglandin
PI: Propidium iodide
PM: Permethrin
PVDF: Polyvinylidene fluoride
ROS: Reactive oxygen species
SDS: Sodium dodecyl sulfate
SDS-PAGE: Sodium dodecyl sulfate–polyacrylamide gel electrophoresis
SOD: Superoxide dismutase
SPs: Synthetic pyrethroids
TBA: Total bile acids
TBARS: Thiobarbituric acid reactive substance
TNF: Tumor necrosis factor
VGSC: Voltage-gated sodium channels
