# Supplementary figures and images for: Inflammatory and cytotoxic effects of bifenthrin in primary microglia and organotypic hippocampal slice cultures
Source: J Neuroinflammation. 2018 May 24;15:159. doi: 10.1186/s12974-018-1198-1 (PMC5968622; doi:10.1186/s12974-018-1198-1)

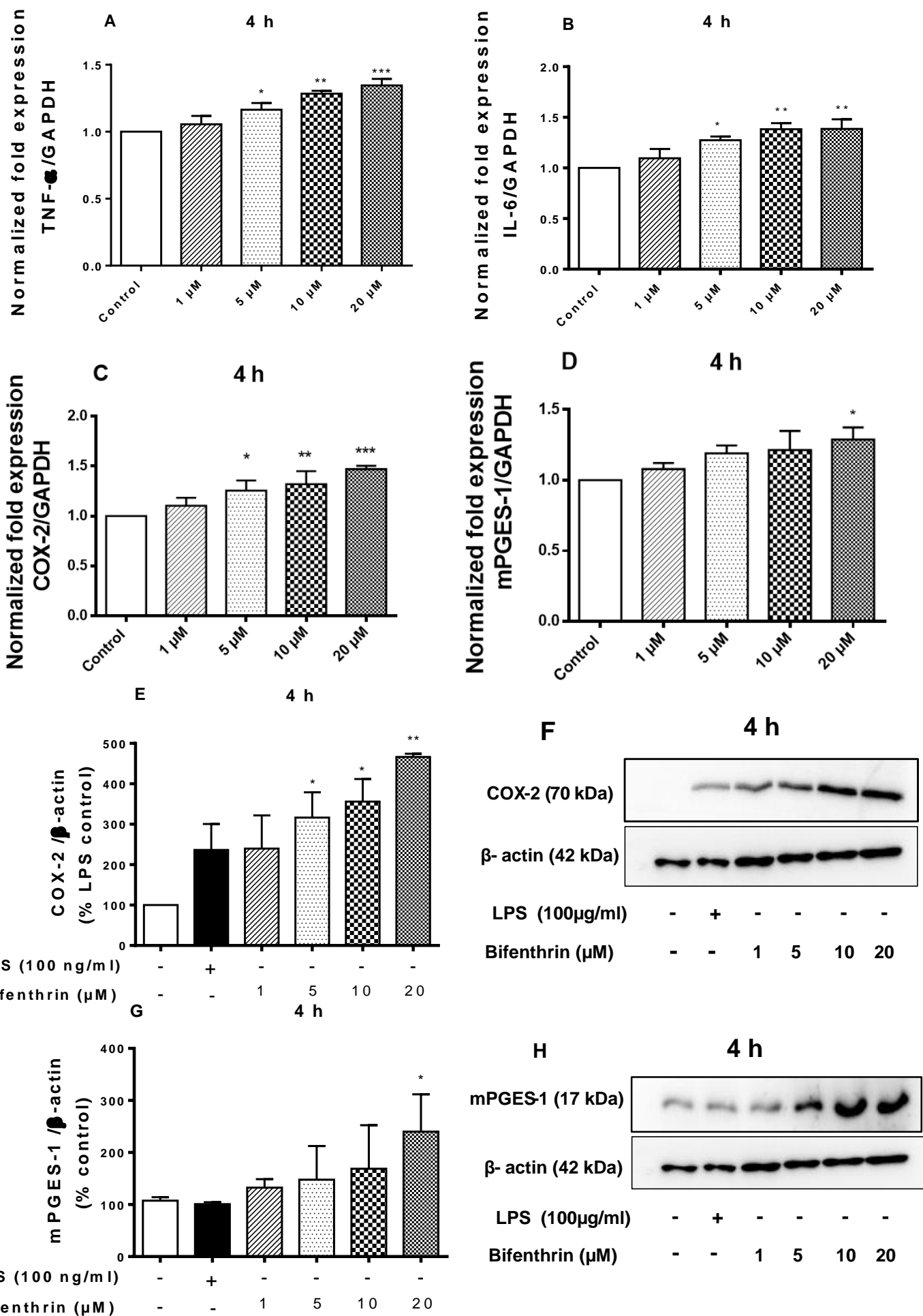

Supplement: Supplementary file 1 — BF exposure increases the expression of pro-inflammatory markers in primary microglia cells. Microglial cells were exposed to different concentrations of BF for 4 h. Gene expression of TNF-alpha (A), IL-6 (B), COX-2 (C), and mPGES-1 (D) was analyzed by real-time quantitative PCR. GAPDH was used as an internal control for normalization, and data were quantified by using the comparative cycle threshold Ct method. Similarly, cells were treated with BF and thereafter incubated with or without LPS (100 ng/mL) as a positive control (black column) for 4 h. Whole cell lysates were subjected to Western blot for COX-2 (E and F), mPGES-1 (G and H), and beta-actin. To confirm equal sample loading, beta-actin was used for normalization. Moreover, data are presented as percentage control of DMSO. Statistical analyses were carried out by using one-way ANOVA followed by post hoc Student–Newman–Keuls test. Results are expressed as means ± SEM of three independent experiments. *p < 0.05; **p < 0.01; **p < 0.001 compared with control (DMSO, white column). (PDF 212 kb) [file 12974_2018_1198_MOESM1_ESM.pdf]

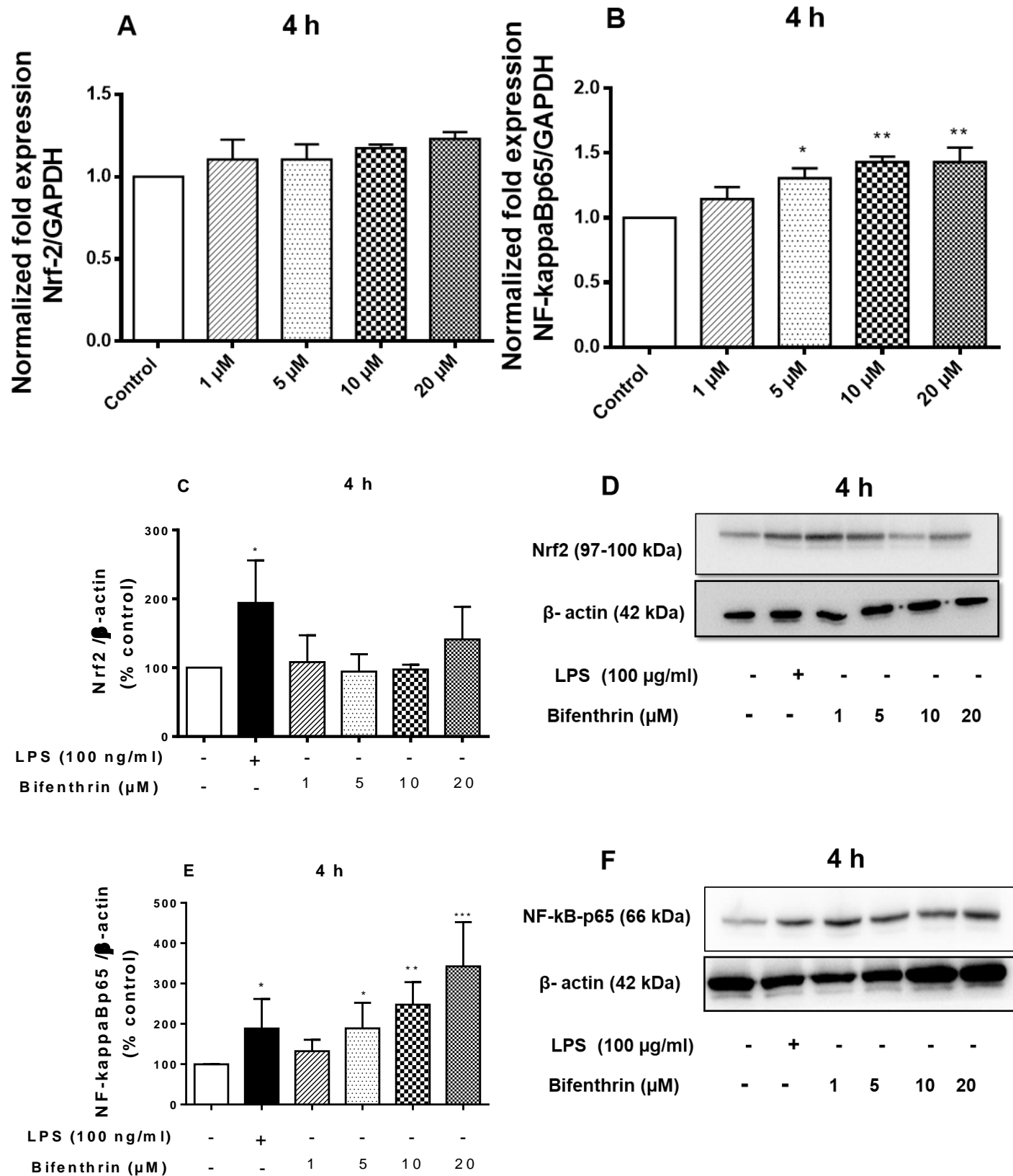

Supplement: Supplementary file 2 — BF exposure increases the expression of Nrf-2 and NF-kappaB in primary microglia. Microglial cells were exposed to different concentrations of BF (1–20 μM) for 4 h. Gene expression of Nrf-2 (A) and NF-kappaBp65 (B) was analyzed by real-time quantitative PCR. GAPDH was used as an internal control for normalization, and data were quantified by using the comparative cycle threshold Ct method. Similarly, cells were treated with BF thereafter incubated with or without LPS (100 ng/mL) as a positive control (black column) for 4 h. Whole cell lysates were subjected to Western blot for Nrf-2 (C and D), NF-kappaBp65 (E and F), and beta-actin. Data are presented as percentage control of DMSO. Statistical analyses were carried out by using one-way ANOVA followed by post hoc Student–Newman–Keuls test. Results are expressed as means ± SEM of three independent experiments. *p < 0.05; **p < 0.01; **p < 0.001 compared with control (DMSO, white column). (PDF 258 kb) [file 12974_2018_1198_MOESM2_ESM.pdf]
